# Homocultology

**Published:** 1873-12

**Authors:** 


Homocultology—An Essay. "Published for the Author," and for sale at the "Progressive Publishing House," New York.

Apropos of Scoptzi in Roumania comes the proposition of Scoptzi in America, in the attractive formula of "homoculture." A few extracts will give a tolerably clear idea of the *gist* of the matter, e. g.: "Original and truthful ideas, from which practical benefits may be realized, are now of more value to mankind than all the creeds and ancient religions ever invented." "Ring out the old and ring in the new." "It follows that all these foolish ceremonies, absurd faiths, and improbable creeds, must be unlearned, either here or hereafter, before any progress can be made."

"*Prevention* is the only word in which there is any power or potency. Cure is but a broken reed! "*Prevention* is the powerful lever which must be used to arrest the increase of bad men, and the propagation of criminals. *First:* we propose the gradual improvement of the human race by studying those relations and adaptations of the physical, mental and moral nature, which will produce perfect children—perfect men and women. *Second:* the prevention of propagation by those who are hopelessly incurable, mentally, morally or physically. *Third:* the emasculation of those who are convicted of high crimes and misdemeanors, and of those who are liable to communicate incurable and loathsome diseases to their offspring."

"These three propositions constitute the Alpha and Omega of all reforms. If they were carried into practice, *no other reforms would be required.*" "For the crimes of rape, murder, arson and highway robbery, the skill of the surgeon should, by law, be called into requisition immediately upon the conviction of the criminal. This punishment would carry more wholesome terror into the minds of thieves and murderers than the death penalty; and if there is any injustice in hanging a man, and thus hurrying him into the presence of his Maker, our plan will not do the felon that injustice. He will have ample time to whiten and save his sinful soul. Capital punishment may be abolished by adopting our method; society will be spared those scenes which take place at the scaffold. The exertions of the embassadors of Christ to purify a criminal, whose organization made him a bad man, will no longer be necessary. If they ever do make a murderer a fit associate for the angels, our plan will suffer him to live as a model for others, and when he does die, we will be sure that he has not left half a score of young murderers to grow up and eclipse their sire upon the record of crime."

"That greater men and women than have yet been born on earth can be produced now, we have every reason to believe. The super-eminent genius of Homer, Shakespeare and Byron may yet be surpassed. The intellect of a Socrates, Webster, Napoleon or Brougham may be again produced. The character and example of Washington may be equaled. Novelists like Dickens, Scott and Dumas will appear again in hundreds. Phidias, Angelo, DeVinci and Delaroche may be duplicated ; and, instead of having one great man and woman in a century, we will have hundreds," &c., &c.

But enough—quite enough—of this *rosy-covered* pamphlet.

